# The utility of magnetic resonance imaging in a trial to assess the effect of renal denervation in heart failure with preserved ejection fraction

**DOI:** 10.1186/1532-429X-17-S1-T7

**Published:** 2015-02-03

**Authors:** Ricardo Wage, Hitesh Patel, Gillian C Smith, Jennifer Keegan, Peter Gatehouse, Vassilis Vassiliou, Ee Ling Heng, Carl Hayward, Stuart D Rosen, Alexander Lyon, Raad Mohiaddin, Sanjay K Prasad, Dudley J Pennell, Carlo Di Mario

**Affiliations:** 1 Royal Brompton Hospital, London, UK; 2 Ealing Hospital, London, UK

## Background

Heart failure with preserved ejection fraction (HF-PEF) is common and has a poor prognosis with a 3 year mortality rate of 23%. There are currently no effective therapies for this condition. HF-PEF is characterized by symptoms of heart failure, normal or a mildly impaired ejection fraction and evidence of adverse cardiac and vascular remodelling. A feature of all heart failure is a heightened sympathetic nervous system (SNS), which can now be abrogated using renal denervation (RD).

## Methods

We are conducting a study of patients with HF-PEF who will be randomised (2:1) to receive RD or open control. As a phase II mechanistic study we are primarily investigating the effect of RD on patient symptoms (questionnaire), cardio-pulmonary exercise function, B-type natriuretic peptide levels, left ventricular filling pressures, left ventricular mass and left atrial volume. We are also assessing macrovascular function using aorta imaging to calculate aorta distensibility, pulse wave velocity and aortic flow. Finally, we will investigate the effect of RD on renal artery blood flow. Cardiac, aorta and renal magnetic resonance image (MRI) sequences will be used to provide this data. Tests will be performed at baseline, three months and 12 months.

## Results

The study is on-going. Currently 25 patients have been randomized. Quantitative MRI will be performed blinded. Examples of baseline data will be presented for illustrative purposes.

## Conclusions

The effects of RD on the heart, vasculature and the kidneys have not been fully elucidated. Using MRI, we aim to clarify some of the mechanisms of action of RD in a population of patients with HF-PEF.

## Funding

NIHR Cardiovascular Biomedical Research Unit, Royal Brompton Hospital, UK.

**Figure 1:**
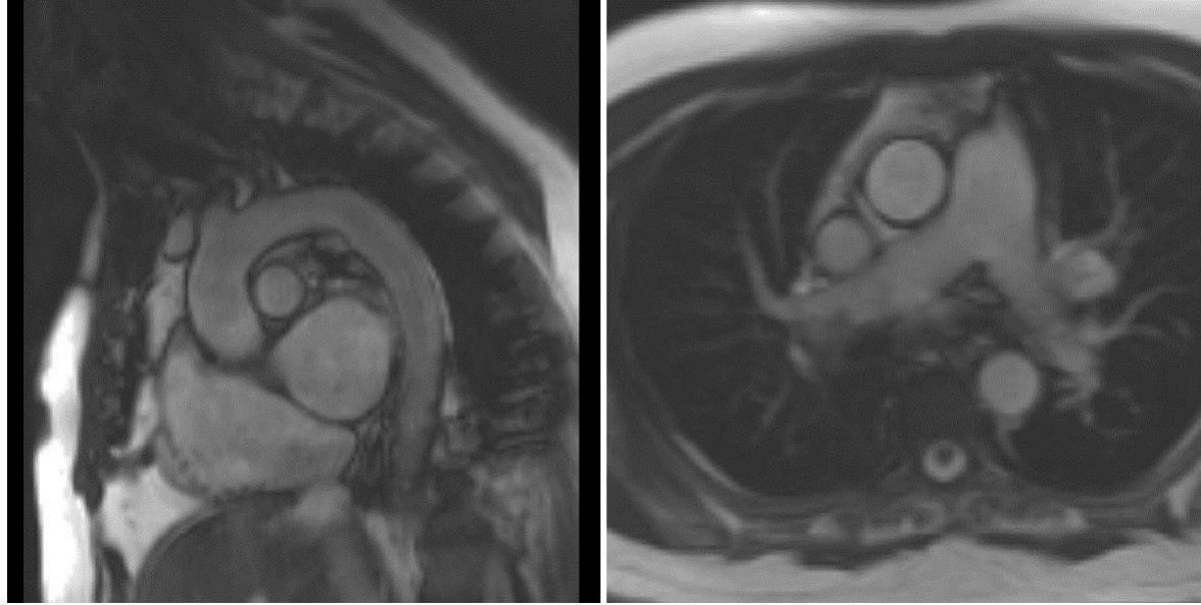
Left image: Trufisp-GRE cine of the aorta and arch.Right image: Through plane of the ascending and descending aorta at pulmonary artery level. This image will be used to plan the aorta distensibility and pulse wave velocity sequences.

**Figure 2:**
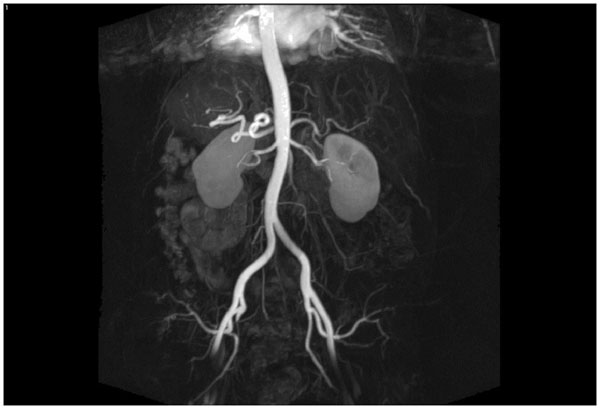
Magnetic resonance angiography of the renal arteries.

